# Poor outcomes of revision total knee arthroplasty in patients with septic loosening compared to patients with aseptic loosening

**DOI:** 10.1186/s13018-021-02766-y

**Published:** 2021-10-18

**Authors:** Ji-Hoon Baek, Su Chan Lee, Hosun Jin, Jin-Woo Kim, Hye Sun Ahn, Chang Hyun Nam

**Affiliations:** 1 Joint & Arthritis Research, Department of Orthopaedic Surgery, Himchan Hospital, 120, Sinmok-ro, Yangcheon-gu, Seoul, Republic of Korea; 2 Department of Orthopaedic Surgery, Nowon Eulji Medical Center, Eulji University, Seoul, Republic of Korea

**Keywords:** Revision, Total knee arthroplasty, Septic, Aseptic, Outcomes

## Abstract

**Background:**

The purpose of this study was to compare the functional outcomes, activity levels, mortalities, implant survival rates, and complications in revision total knee arthroplasty (TKA) of patients with septic loosening with those in patients with aseptic loosening over a minimum 10-year follow-up period.

**Methods:**

A cohort of 78 patients (36 septic loosening and 42 aseptic loosening) was selected between January 2008 and December 2009. The functional outcomes, activity levels, mortalities, implant survival rates, and complications of revision TKA in patients with septic and aseptic loosening were compared.

**Results:**

The mean Knee Society knee scores in the septic and aseptic groups improved from 36.7 and 37.4 preoperatively to 65.3 and 76.8 points at the final follow-up, respectively (*p* < 0.05). Outdoor ambulatory patients at the final follow-up included 20 of 29 (69.0%) patients in the septic group and 35 of 39 (89.7%) patients in the aseptic group (*p* < 0.05). The cumulative mortality rates in the septic and aseptic groups were 19.4% (7/36) and 7.1% (3/42) (*p* = 0.102) at final follow-up, respectively. Kaplan–Meier survivorship analysis with re-revision of either component as an endpoint in the septic and aseptic groups estimated 86.5% and 95.5% chance of survival for 10 years, respectively.

**Conclusions:**

Revision TKA in patients with septic loosening had worse functional outcomes and higher mortality over a minimum 10-year follow-up period compared with that in patients with aseptic loosening.

**Level of evidence:**

IV.

## Introduction

Total knee arthroplasty (TKA) is an effective surgical intervention for treatment of late-stage osteoarthritis of the knee by reducing pain and improving function. With increased demand for primary TKA in the elderly population, there is a concomitant expected increase in revision TKA [1, 2]. The main causes of failure following TKA are aseptic loosening, infection, component malposition, instability, and stiffness [1, 3–5]. The need for revision TKAs showed a rapid 267% increase between 2001 and 2010 in Korea [6]. The increase in revision TKA use can be explained by the increasing aging population and improved accessibility to the healthcare system [7].

The burden of revision TKA is substantially greater and requires additional hospitalization relative to that of primary surgery. Results following revision TKA are less predictable than after the primary procedure [8], and it is reasonable to expect that outcomes such as survival and complication rate following revision TKA will be inferior to those following the primary surgery [9]. With an increase in number of revision TKA surgeries, greater understanding of long-term results is required. Although several studies have reported on revision TKA, limited data are available on the long-term follow-up outcomes of revision TKA in patients required due to either septic or aseptic loosening.

Here, we designed a retrospective observational study to compare the functional outcomes, activity levels, mortalities, implant survival rates, and complications of revision TKA in patients with septic or aseptic loosening over a minimum 10-year follow-up period.

## Materials and methods

**Fig. 1:**
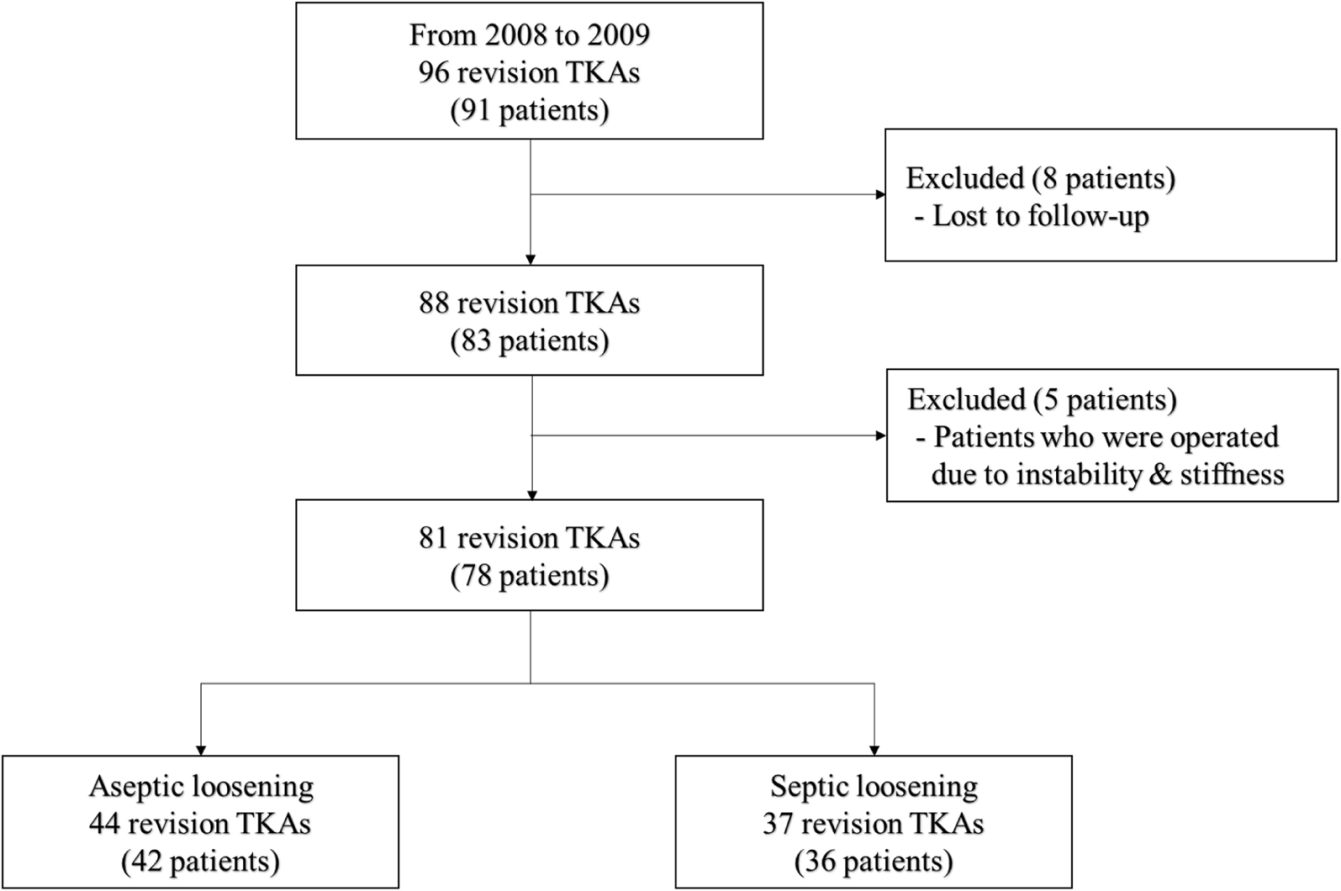
A schematic showing selection of subjects for this study

The design and protocol of this retrospective study were approved by the Institutional Review Board of our hospital. The requirement of informed consent was waived due to the retrospective nature of the study. Between January 2008 and December 2009, a consecutive series of 96 revision TKAs was performed in 91 patients using a single implant system at our hospital. Patients receiving a partial revision, a hinged arthroplasty, revision of unicondylar arthroplasty, isolated polyethylene exchange, and/or patellar resurfacing were not included. Patients were allocated to 1 of 4 categories: aseptic loosening (49 knees/47 patients), septic loosening (40 knees/39 patients), instability (6 knees/4 patients), and severe stiffness (1 knee/1 patient). In our cohort, aseptic loosening was the most common cause of failure, followed by infection, instability, and then stiffness. Of these 91 patients, eight (8 knees) were excluded from the study due to follow-up loss, and five (7 knees) with instability and severe stiffness were excluded due to low number. The final aseptic cohort consisted of 37 females (39 knees) and five males (5 knees), with an average age at surgery of 67.2 years (range 43 to 80 years). The septic group included 30 females (31 knees) and six males (6 knees) with an average age at surgery of 67.5 years (range 37 to 78 years) (Fig. 1). Diagnosis of infection for the septic group was confirmed through aspirated joint fluid analysis, which is performed via microbiological culture, demonstration of acute inflammation by periprosthetic tissue, a WBC count of > 2000/μL, or a granulocytes percentage of > 70% in the joint fluid [10]. Joint fluid was obtained preoperatively and intraoperatively to confirm the presence of causative organisms. Demographic data of sex, age, initial diagnosis, body mass index, pre-operative Koval category [11], and pre-operative Knee Society Score [12] were obtained by reviewing medical records (Table 1). The mean follow-up period was 11.8 years (range 10.2–14.0 years) in the aseptic group and 11.8 years (range 10.5–14.0 years) in the septic group.

**Table 1:** Demographics of patients

	Septic group	Aseptic group	*p-*value
Number of patients	36	42	
Number of TKA	37	44	
Male: female	6: 30	5: 37	0.531
Age (years) (mean ± SD)	67.5 ± 8.1	67.2 ± 7.5	0.862
Body mass index	25.2 ± 2.8	27.4 ± 3.7	0.003
Diagnosis			
Aseptic loosening		42	
Septic loosening	36		
Koval grade by pre-operative status			0.971
I	30	35	
II	6	7	
III–VI	0	0	
Pre-operative			
Knee Society knee score	36.7 ± 5.3	37.4 ± 5.2	
Function score	35.5 ± 6.2	36.7 ± 6.4	

TKA, total knee arthroplasty; SD, standard deviation

All surgical procedures were performed by three senior surgeons using standardized instrumentation and uniform surgical technique. Two-stage reimplantation was performed in all septic group patients. The first stage involved removal of the component and cement spacer implantation. The second stage involved implantation of the new components in accordance with adequate infection control. All patients were treated using the Scorpio Total Stabilizer Revision Knee System (Stryker Orthopaedics, Mahwah, NJ, USA). All implants were inserted with the Simplex P bone cement (Stryker Orthopaedics, Mahwah, NJ, USA). Patients were mobilized with immediate weight-bearing as tolerated, and active exercise was initiated under supervision of a physiotherapist. Patients underwent clinical and radiographic follow-up at post-operation two and six weeks; three, six, nine, and 12 months; and annually thereafter. During follow-up evaluations, patients who did not return for scheduled visits were contacted by telephone. Two nurses and one private doctor found and visited non-responders. Clinical evaluations were performed using the Knee Society rating system [12]. Results were classified as excellent (80–100), good (70–79), fair (60–69), or poor (< 60). Change of patient activity and mortality were compared within a minimum of 10 years between the two groups. Activity level was defined as follows: I, independent community ambulatory; II, community ambulatory with cane; III, community ambulatory with walker/crutches; IV, independent household ambulator; V, household ambulatory with cane; VI, household ambulatory with walker/crutches, and VII, nonfunctional ambulatory [11]. In the analysis, Koval grades I, II, and III cases were classified as outdoor ambulatory patients who can walk outside, whereas Koval grades IV, V, VI, and VII cases were classified as shut-in patients who walk only at home. Radiographic analysis included long-leg standing radiography from the pelvis to the ankle joint for evaluating the axis, weight-bearing anteroposterior view, non-weight bearing anteroposterior view, lateral view at 30° flexion, and skyline view of the patella. Each radiograph was assessed using the Knee Society evaluation system [13]. Mortality status and implant survival analysis were identified using hospital records and/or by interviews with family members. Patients unable to attend follow-up evaluations were interviewed by telephone. During the follow-up evaluations, the same caregiver previously interviewed during the patient’s hospitalization was questioned.

Cumulative crude mortality rate was calculated at three, six, and 10 years and compared between the two groups. Kaplan–Meier survival analysis was performed for both groups for a minimum 10-year follow-up period, using mortality as a primary end point. Additionally, Kaplan–Meier survival analysis was performed for all knees for a minimum 10-year follow-up period with re-revision of either component as an endpoint. The correlation of survival rates in the groups was tested using the log-rank test. Student’s *t*-test was used to analyze age and body mass index, and Mann–Whitney test was used to analyze Koval grade. The chi square test was used to analyze sex, Knee Society knee and function scores, and cumulative mortality. The analyses were carried out using IBM SPSS Statistics software version 18.0 (IBM, Armonk, NY, USA). All reported *p*-values were two-sided, and *p* < 0.05 was considered statistically significant.

## Results

The mean Knee Society knee scores in the septic and aseptic groups improved from 36.7 and 37.4 points preoperatively to 65.3 and 76.8 points at the final follow-up, respectively (*p* < 0.05). Clinical outcomes were classified as excellent or good for 17 patients (17/29, 58.6%), fair for two, and poor for 10 in the septic group and excellent or good for 30 patients (30/39, 76.9%), fair for five, and poor for four in the aseptic group. Mean preoperative function scores in the septic and aseptic groups improved from 35.5 and 36.7 points to 62.5 and 73.1 points at the final follow-up, respectively (*p* < 0.05). Functional outcomes were classified as excellent or good for 17 patients (17/29, 58.6%), fair for three, and poor for nine in the septic group and excellent or good for 30 patients (30/39, 76.9%), fair for five, and poor for four in the aseptic group.

**Fig. 2:**
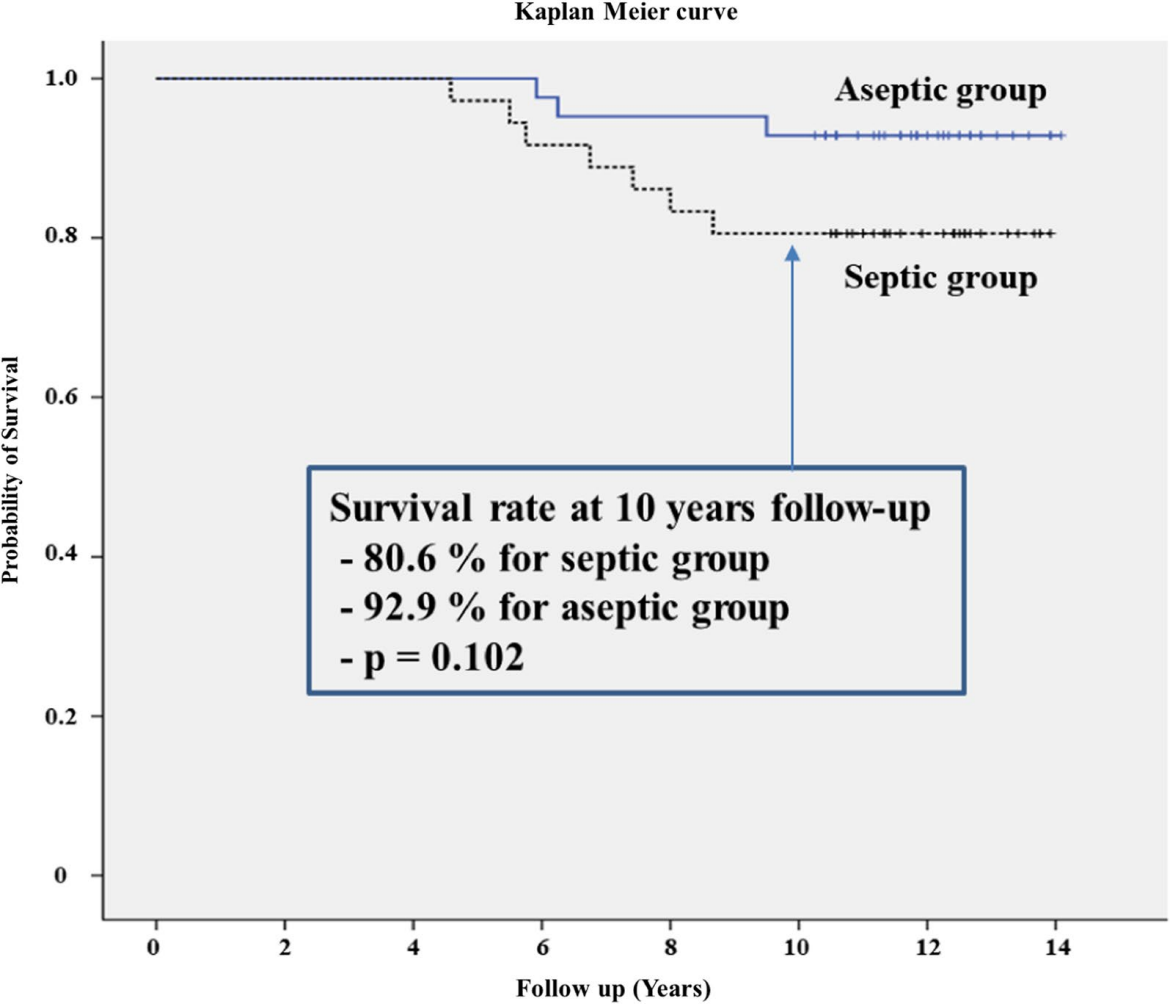
Kaplan–Meier survival analysis of mortality comparing the septic group (black) and aseptic group (blue)

Among 29 surviving patients in the septic group at a minimum follow-up period of 10 years, 20 were outdoor ambulatory, and nine were shut-in patients (Table 2). However, among 39 surviving patients in the aseptic group, 35 were outdoor ambulatory, and four were shut-in patients (Table 3). The cumulative mortality rates in septic and aseptic groups were 19.4% (7/36) and 7.1% (3/42) at the final follow-up, respectively (*p* = 0.102) (Fig. 2 and Table 2).

**Table 2:** Comparison of mortality, Koval grade, and Knee Society Score between septic and aseptic groups

	Septic group	Aseptic group	*p-*value
3 years	0/36 (0%)	0/42 (0%)	
6 years	3/36 (8.3%)	1/42 (2.4%)	
10 years	7/36 (19.4%)	3/42 (7.1%)	
Mortality at final f/u	7/36 (19.4%)	3/42 (7.1%)	0.102
Koval grade at final f/u			< 0.05
I	13	26	
II	4	4	
III	3	5	
IV	6	3	
V	2	0	
VI	1	0	
VII	0	1	
Post-operative			
Knee Society knee score	65.3 ± 21.1	76.8 ± 15.6	< 0.05
Function score	62.5 ± 20.2	73.1 ± 17.2	< 0.05

f/u: follow up

**Table 3:** Comparison of mortality between studies

References	Number of patients	Mean follow-up (year)	Mortality	Other
Choi and Bedair [18]	88 (septic) 88 (aseptic)	4	18% (septic) 3% (aseptic)	
Yao et al. [22]	1370 (septic) 1740 (aseptic)	10	47% (septic) 34% (aseptic)	15 years: 73% (septic) 60% (aseptic)
Matar et al. [23]	309 (septic) 945 (aseptic)	10	31.3% (septic) 19.8% (aseptic)	17 years: 33.9% (septic) 25.0% (aseptic)
Present study	36 (septic) 42 (aseptic)	11.8	19.4% (septic) 7.1% (aseptic)

A non-progressive radiolucent line (radiographic demarcation ≥ 2 mm) was observed in two femoral components and three tibial components in the septic group and two femoral and four tibial components in the aseptic group during serial follow-up. All femoral and tibial components in both groups were well fixed without loosening, and no obvious tibial insert polyethylene wear was observed at the final follow-up.

**Fig. 3:**
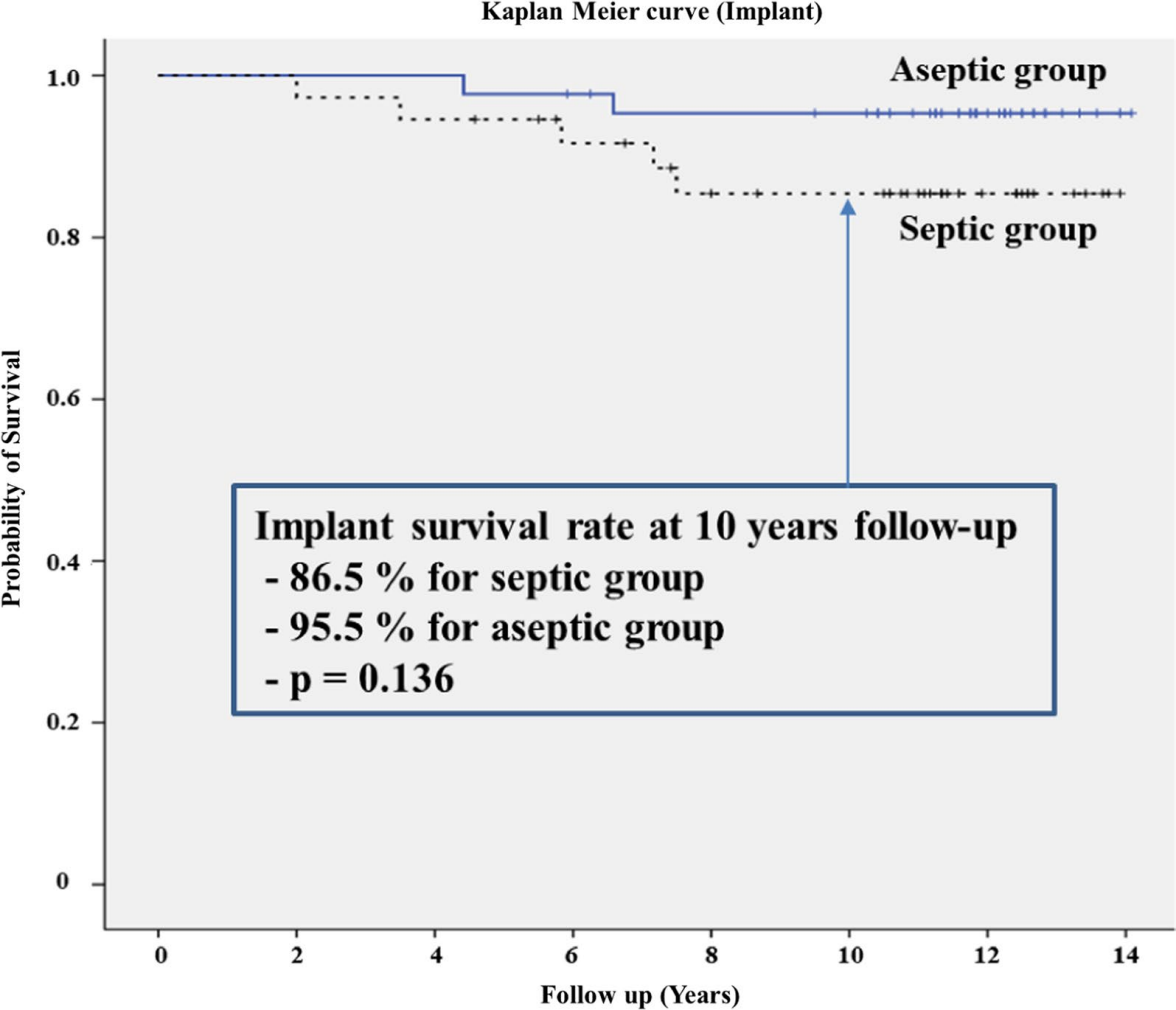
Kaplan–Meier survival analysis of implant comparing the septic group (black) and aseptic group (blue)

Regarding postoperative complications, periprosthetic joint infections were observed in five and two patients in the septic and aseptic groups, respectively. All patients were treated with a two-stage revision procedure. No revision surgery was needed for any other reason such as aseptic loosening, component malalignment, liner wear, instability, or stiffness. Periprosthetic fracture of the femoral shaft was observed in one and two patients in septic and aseptic groups, respectively. All patients were treated with plate and screw fixations. Kaplan–Meier survivorship analysis with re-revision of either component as an endpoint in the septic and aseptic groups was estimated at 86.5% (95% confidence interval 83.3% to 89.7%) and 95.5% (95% confidence interval 92.3% to 98.7%) chances of 10-year survival, respectively (Fig. 3).

## Discussion

This study aimed to compare the functional outcomes, activity levels, mortalities, implant survival rates, and complications of revision TKA in a contemporary cohort of patients with septic loosening compared to aseptic loosening at a minimum of 10 years of follow-up. The most important finding of the present study was that revision TKA in patients with septic loosening showed worse functional outcomes and higher rates of mortality and revision at a minimum of 10 years follow-up.

Prediction of functional outcome after revision TKA might provide insights to better manage patient expectations. Meek et al. [14] reported that revision surgery for infection was associated with reasonable function and satisfaction scores at a mean follow-up of 3.4 years. Patil et al. [15] reported that patients undergoing revision for an infected TKA had better functional outcomes at a mean follow-up of 3.3 years compared to those with revision for aseptic reasons. However, it is difficult to evaluate the long-term outcomes from these studies because of the short-term follow-up periods. Other studies reported that the results of revision TKA after infection were less favorable than those of revision TKA after aseptic reasons [5, 16, 17]. In this study, at a minimum of 10 years follow-up, revision TKA in patients with septic loosening remained associated with worse functional outcomes. It is reasonable to conclude that patients with infected TKA might have a limited range of motion and experience more pain after two-stage reimplantation procedures which may affect knee function.

In this study, the 19.4% mortality rate after TKA in patients with septic loosening was higher than the 7.1% of patients with aseptic loosening at a minimum follow-up period of 10 years. The higher mortality rate observed in the septic group is consistent with the study reported by Choi and Bedair [18], who reported that mortality after septic revision was six-fold higher than that of aseptic revision (18% vs. 3%) at a mean follow-up of four years. Although direct comparison of mortality between studies is difficult because of differences in demographic data such as age, sex, medical comorbidity, and ethnic difference, mortality after revision TKA in patients with septic loosening is higher than that of patients with aseptic loosening (Table 3).

The present study showed that the re-revision rate due to recurrence of infection was higher in septic than in aseptic patients. The incidence of infection following primary TKA has a reported range of 1% to 2% [19]; however, reinfection rates after two-stage revision procedures occurred in up to 19% of surgeries [20, 21]. These findings correspond with outcomes of this study for long-term follow-up. The rates of re-revision in our study were 13.5% in septic patients and 4.5% in aseptic patients over 10 years.

This study has several limitations. First, our study was retrospective and performed in a cohort of prospectively followed patients. Second, because few patients underwent revision TKA at a single center, the results might not be generalizable. Third, in the mortality rate analysis, consideration for other medical comorbidities was scarce, which can substantially impact mortality in elderly populations. Therefore, we were not sure whether infection itself contributed to mortality rates or whether medical comorbidities were related to mortality rates. Fourth, we did not include patients with re-surgery due to instability and stiffness in the analysis considering that low numbers would limit the accuracy of comparison. Lastly, this study was not a single-surgeon series; however, all surgeons were high-volume knee surgeons and used the same surgical technique and consistent perioperative protocols. The strength of this study is in the long-term clinical outcomes after revision TKA in patients with septic loosening compared with aseptic loosening.

In conclusion, revision TKA in patients with septic loosening showed worse functional outcomes and higher mortality at a minimum follow-up period of 10 years compared to that in patients with aseptic loosening. Therefore, patients with septic loosening should be counseled appropriately before revision surgery so that they have reasonable expectations about post-surgery outcomes.

## Data Availability

All data generated or analysed during this study are included in this published article.

## Abbreviations

TKA: Total knee arthroplasty
SD: Standard deviation
f/u: Follow up
